# Thyroid Response to Peripheral Endocrine Factors: Neuropeptide Y Influences Thyroid Function in the Reptile *Podarcis siculus*

**DOI:** 10.3390/ijms262311513

**Published:** 2025-11-27

**Authors:** Rosaria Sciarrillo, Assunta Lallo, Francesca Carrella, Vito Gallicchio, Aldo Mileo, Benedetta Sgangarella Valvano, Maria De Falco

**Affiliations:** 1 Department of Science and Technologies, University of Sannio, Via F. de Sanctis snc, 82100 Benevento, Italy; alallo@unisannio.it (A.L.); fcarrella@unisannio.it (F.C.); 2 Vascular Surgery, Hospital of National Importance San Giuseppe Moscati, Via Contrada Amoretta, 83100 Avellino, Italy; vitogallicchio@gmail.com; 3 Department of Medicine and Health Sciences, University of Molise, 86100 Campobasso, Italy; aldomileo@unibas.it; 4 Department of Biology, University of Naples “Federico II”, 80126 Naples, Italy; bvalvano@gmail.com (B.S.V.); madefalco@unina.it (M.D.F.); 5 National Institute of Biostructures and Biosystems (INBB), 00136 Rome, Italy

**Keywords:** neuropeptide Y, hypothalamic–pituitary–thyroid axis, thyroid gland, thyroid hormones, lizard

## Abstract

Neuropeptide Y (NPY) is a small signalling molecule produced by neurons through the cleavage of a precursor protein. It generally binds to and activates G protein-coupled receptors to modulate complex homeostatic processes and behaviours in animals. Mammals provide definitive proof of the role of NPY in the thyroid axis, but in reptiles, this link is unclear. We demonstrate that the thyroid axis responds to NPY administration in a dose-dependent manner, with a reduction in plasma TRH and TSH concentrations, and an increase in plasma T_3_ and T_4_ levels 2 and 24 h after administration, suggesting that NPY may activate the thyroid axis. This increase in thyroid hormones is supported by morphological findings in the thyroid gland, which show clear signs of stimulation demonstrated by a dose-dependent increase in the height of the follicular epithelium and the presence of numerous resorption vacuoles. Moreover, we investigated the 5-T_4_ ORD (type II) Monodeiodinase activity at the hepatic level, showing that NPY increased hepatic T_3_ levels and decreased hepatic T_4_ levels, and suggesting an alternative mode of signalling by NPY on peripheral biosynthesis of thyroid hormones. Our study helps to address the current lack of research in the field of endocrinology concerning the effects of NPY on metabolism and thyroid function.

## 1. Introduction

Neuropeptides are small signalling molecules produced by neurons from proneuropeptides. They bind to and activate G-protein-coupled receptors, thereby modulating complex homeostatic mechanisms and behaviour in animals [1,2,3,4,5,6,7,8,9,10].

Neuropeptide Y (NPY) belongs to the pancreatic polypeptide family. So do peptide YY (PYY) and pancreatic polypeptide (PP). This family was first isolated from pig brains in 1982 [11]. NPY is considered the most conserved peptide among vertebrate species; it is thought that non-mammalian species share a common ancestor that diverged from mammals in their NPY sequence [10,12].

The main source of NPY in the central and peripheral nervous systems is the arcuate nucleus [5,8,13,14], although NPY has also been shown to be highly expressed in the adrenal glands, white adipose tissue, and bone, among other tissues, indicating the potential involvement of this neuropeptide in a wide range of physiological responses, including adipogenesis, regulation of bone mass and energy metabolism, locomotion, anxiety, learning and memory, epilepsy, circadian rhythm, and cardiovascular function [15,16,17,18,19,20,21,22].

NPY is considered a potent orexigenic neuropeptide, a major regulator of food intake, energy homeostasis, and appetite in several species [23,24]. It is also possible that different levels of NPY will directly influence the hypothalamic–pituitary–thyroid axis by regulating thyrotropin-releasing hormone (TRH). In rats, NPY stimulates energy balance by suppressing TRH [25] and stimulating thyroid follicular cells, and modulates pituitary TSH secretion. In mammals, thyroid hormones can, in turn, regulate NPY expression through the activation/inactivation of AMP-activated protein kinase (AMPK) [26]. In fish, there are a few published studies examining the association between appetite regulatory peptides and the thyroid axis [27,28].

There are very few studies on the role and regulation of NPY in reptiles in relation to their phylogenetic and ecological importance. Reptiles were the first organisms to adapt to a terrestrial way of life. Despite the richness of the species, research on reptiles is significantly less extensive than research on mammals [29], highlighting a missed opportunity for comparative and evolutionary study. The order Squamata, which includes snakes and lizards, represents approximately 96.78% of all reptile species; lizards alone constitute 62.9% of Squamata diversity [30]. Furthermore, a sophisticated and complex mechanism of regulation of the hypothalamic–pituitary–thyroid axis similar to that of mammals has been identified in Squamata [31], fueling further interest in exploring the regulation of their functions, especially in the regulation of thyroid function. Furthermore, the wall lizard, *Podarcis siculus*, has its own annual thyroid cycle, divided into four different phases: recrudescent (March–May) and active (June–July), regressive (August–October), and stasis (November–January) [31], which makes it suitable for the study of the factors responsible for the regulation of endocrine function.

As NPY is found at the crossroads of nutrition, metabolism, and homeostasis, this neuropeptide shows promise as a regulatory agent for thyroid function via the hypothalamic–pituitary–thyroid axis in the lizard *Podarcis siculus*. It works in conjunction with other peripheral endocrine factors, including leptin [32], substance P [33], galanin [34] and endothelin-1 [35]. The finding of NPY-IR fibres around the follicles and the presence of NPY-specific immunoreactivity in the apical parts of the thyrocytes of the lizard *Podarcis siculus* [36] support this.

This study is the first to investigate the effects of neuropeptide Y (NPY) on the morphophysiology of the thyroid gland in *Podarcis siculus* lizards following its intraperitoneal administration. The study evaluates both the central control of the hypothalamic–pituitary–thyroid axis and the peripheral regulation of thyroid hormones in the liver.

## 2. Results

### 2.1. Effects of NPY Administration on Circulating Serum Levels of TRH and TSH

**Figure 1:**
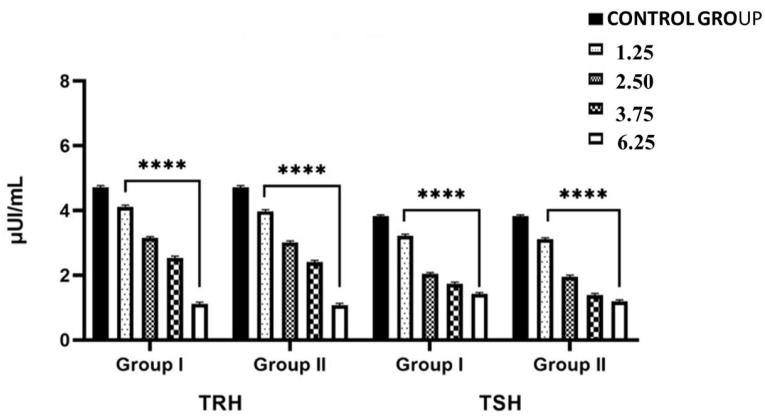
Plasma TRH (μUI/mL), TSH (μUI/mL) levels in *Podarcis siculus* subjected to 1 intraperitoneally injection of 1.25–2.50–3.75–6.25 nmol/100 g body wt of NPY and sacrificed 2 h and 24 h after the injection. (**** *p* < 0.001, in the comparison with the control). A more detailed description is in the text (see Section 4).

Overall changes in circulating serum levels of hypothalamic TRH and pituitary TSH were explained by the interactive effect of dose and experimental time of NPY administration in lizards *Podarcis siculus*. The levels of TRH and TSH in lizards treated with the lowest dose of NPY (1.25 nmol/100 g body wt and were sacrificed 2 h after the injection) were significantly lower compared with the control group (*p* < 0.001). In fact, TRH level decreased significantly from 4.72 ± 0.02 (Group C) to 4.12 ± 0.08 μUI/mL and plasma TSH level decreased significantly from 3.82 ± 0.05 (Group C) to 3.22 ± 0.05 μUI/mL (Figure 1). A further decrease in TRH was also observed in specimens treated with the same dose of NPY but sacrificed 24 h after the last injection (Figure 1). Specifically, TRH levels reached the values of 3.98 ± 0.05 μUI/mL (*p* < 0.001) and TSH 3.11 ± 0.03 μUI/mL (*p* < 0.001) (Figure 1). The greatest inhibition of circulating TRH and TSH levels was found in animals treated with the maximum dose of NPY and sacrificed 24 h after administration (6.25 nmol/100 g body wt) (TRH: 1.11 ± 0.01 μUI/mL vs. 4.72 ± 0.02—control group; *p* < 0.001; TSH: 1.42 ± 0.01 vs. 3.82 ± 0.05 μUI/mL—control group; *p* < 0.001) (Figure 1).

### 2.2. Effects of NPY Administration on Circulating Serum Levels of Thyroid Hormones

**Figure 2:**
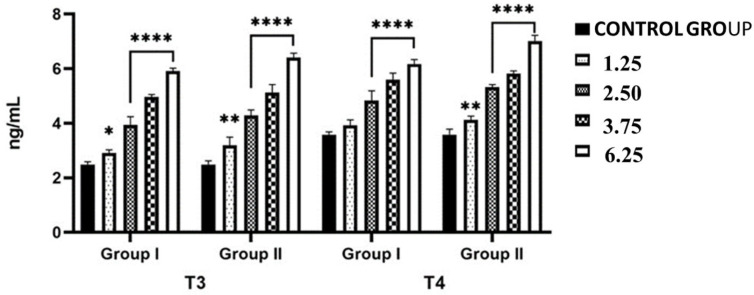
Plasma T_4_ (ng/mL), T_3_ (ng/mL) levels in *Podarcis siculus* subjected to 1 intraperitoneally injection of 1.25–2.50–3.75–6.25 nmol/100 g body wt of NPY and sacrificed 2 h and 24 h after the injection. (* *p* < 0.05, ** *p* < 0.01, **** *p* < 0.001, in the comparison with the control). A more detailed description is in the text (see Section 4).

Both the amount of NPY administered and the timing of administration influenced circulating levels of total thyroid hormone (triiodothyronine, T_3_; thyroxine, T_4_) (Figure 2). Lizards injected with the highest dose of NPY (6.25 nmol/100 g body wt) and sacrificed 24 h after administration had higher serum levels of T_4_ (7.02 ± 0.02 ng/mL) and T_3_ (6.42 ± 0.05 ng/mL) than lizards injected with saline [T_4_ (3.58 ± 0.07 ng/mL); T_3_ (2.49 ± 0.04 ng/mL) *p* < 0.001] (Figure 2). Even only 2 h after administration of the highest dose of NPY, circulating plasma levels of T_4_ and T_3_ were significantly (*p* < 0.001) increased compared to the values of the control specimens (T_4_: 6.18 ± 0.04 ng/mL vs. 3.58 ± 0.07 ng/mL; T_3_: 5.92 ± 0.05 ng/mL vs. 2.49 ± 0.04 ng/mL), but with slightly lower values compared to the lizards treated with the same dose but sacrificed 24 h after the administration (Figure 2).

The same increase in plasma T_4_ and T_3_ concentrations was also found in lizards treated with other doses of NPY; in fact, at the lowest dose of NPY (1.25 nmol/100 g body wt) and sacrificed after 24 h, plasma T_4_ and T_3_ concentrations were significantly higher than those of control lizards (T_4_: 3.92 ± 0.04 ng/mL vs. 3.58 ± 0.07 ng/mL; T_3_: 2.92 ± 0.05 ng/mL vs. 2.49 ± 0.04 ng/mL, *p* < 0.001) (Figure 2).

### 2.3. Effects of NPY Administration on the Hepatic Expression Levels of 5-T_4_ ORD (Type II) Monodeiodinase Activity and T_4_ and T_3_ Contents

**Figure 3:**
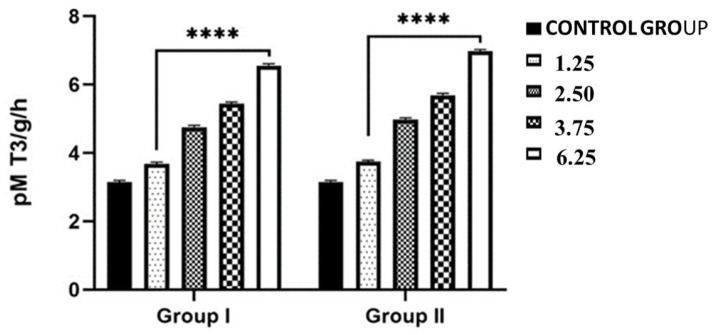
Hepatic 5′-T4 ORD-monodeiodinase (type II) activity (pM T_3_/g/h) in *Podarcis siculus* subjected to one intraperitoneally injection of 1.25–2.50–3.75–6.25 nmol/100 g body wt of NPY and sacrificed 2 h and 24 h after the injection. (**** *p* < 0.001, in the comparison with the control). A more detailed description is in the text (see Section 4).

In the liver, 5-T_4_ ORD (type II) monodeiodinase activity levels showed a significant interaction between the dose and timing of NPY administration (*p* < 0.001). As Figure 3 shows, there was a big rise in the level of hepatic 5′-T_4_ ORD (type II) monodeiodinase activity after treatment with all four doses of NPY after both 2 and 24 h. Indeed, an increase in 5′-T_4_ ORD (type II) activity was observed in lizards exposed to an injection of NPY and sacrificed 2 h after receiving either the lowest dose (1.25 nmol/100 g body weight) (3.68 ± 0.02 pmol T_3_/g/h) or the highest dose (6.25 nmol/100 g body weight) (6.56 ± 0.01 pmol T_3_/g/h), compared to control lizards (3.15 ± 0.05 pmol T_3_/g/h) (Figure 3). The increase in 5′-T_4_ ORD (type II) activity in all specimens treated with the different doses of NPY was found mostly in specimens sacrificed after 24 h from administration; in fact, in lizards treated with the maximum dose of NPY (6.25 nmol/100 g body wt) and sacrificed after 24 h, the activity reached its highest value (6.98 ± 0.05 pM T_3_/g/h) (Figure 3). Furthermore, both after 2 h and 24 h, NPY treatment caused a significant increase in enzymatic activity even in samples treated with the other intermediate doses of NPY (Figure 3).

**Figure 4:**
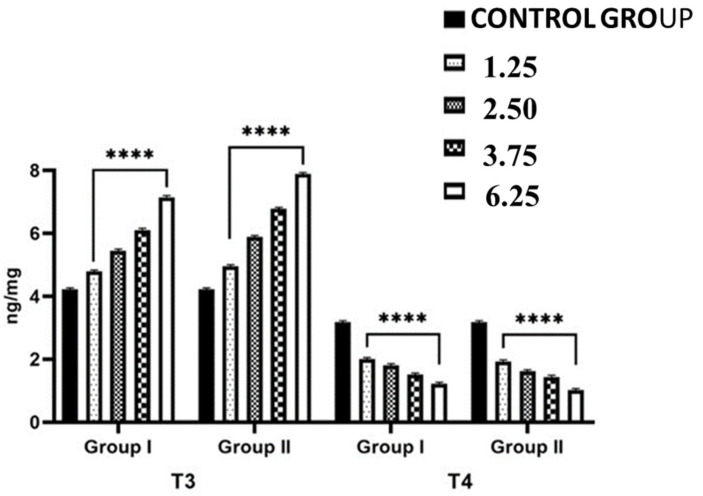
Hepatic T_3_ (ng/mg) and T_4_ (ng/mg) content in *Podarcis siculus* subjected to one intraperitoneally injection of 1.25–2.50–3.75–6.25 nmol/100 g body wt of NPY and sacrificed 2 h and 24 h after the injection. (**** *p* < 0.001, in the comparison with the control). A more detailed description is in the text (see Section 4).

Consistent with the increase in 5′-T_4_ ORD (type II) activity after NPY administration, significant increases (*p* < 0.001) in hepatic T_3_ content and significant (*p* < 0.001) decreases in hepatic T_4_ content were found (Figure 4). Hepatic T_3_ content increase reached from 4.22 ± 0.04 ng/mg (control group) to 4.95 ± 0.01 ng/mg in lizards treated with the lowest dose of NPY sacrificed after 24 h (*p* < 0.001) and 6.89 ± 0.02 ng/mg in lizards treated with the highest dose of NPY sacrificed after 24 h (*p* < 0.001) (Figure 4). On the contrary, T_4_ hepatic contents decreased in all treated groups, reaching the minimum value of 1.02 ± 0.02 ng/mg in lizards treated with the highest dose of NPY sacrificed after 24 h (*p* < 0.001) compared with control specimens (3.18 ± 0.05 ng/mg) (Figure 4). In lizards sacrificed both 2 h and 24 h after the injection of the different doses of NPY, there was the same trend in the activity of the 5′-T_4_ ORD (type II), with a decrease in the hepatic T_4_ content resulting in an increase in hepatic T_3_ (Figure 4).

### 2.4. Effects of NPY Administration on Morphology of Thyroid Gland

**Figure 5:**
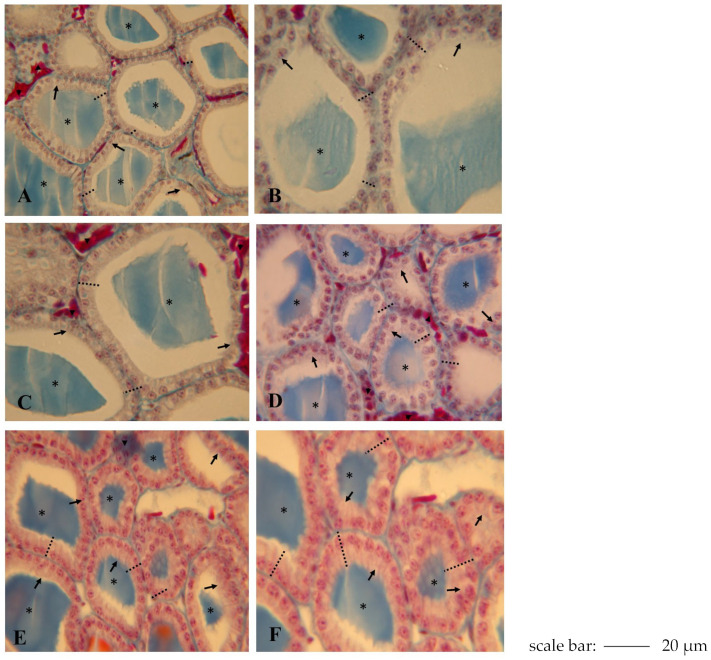
Thyroid gland of lizard *Podarcis siculus* (stain Galgano I). (**A**) Control lizard: the thyroid follicles showed a medium follicular epithelium and the colloid present numerous reabsorbing vacuoles. (**B**) Specimen treated with one intraperitoneally injection of 1.25 nmol/100 g body weight of NPY and sacrificed 24 h after the injection (Group II): the cuboidal follicular epithelial cells, the colloid, and reabsorption vacuoles are shown. (**C**) Specimen treated with 1 intraperitoneally injection of 2.50 nmol/100 g body weight of NPY and sacrificed 24 h after the last injection (Group II): the follicular epithelium is high, and the colloid is present in the follicles with an evident vascularization of the gland. (**D**) Specimen treated with one intraperitoneally injection of 3.75 nmol/100 g body weight of NPY and sacrificed 24 h after the injection (Group II): the thyrocytes assume a cubic shape and the colloid showed many resorption vacuoles. (**E**) Specimen treated with one intraperitoneally injection of 6.25 nmol/100 g body weight of NPY and sacrificed 24 h after the injection (Group II): the follicular epithelium is still higher than normal, the colloid present an evident reabsorbing vacuoles. (**F**) Specimen treated with one intraperitoneally injection of 6.25 nmol/100 g body weight of NPY and sacrificed 2h after the injection (Group I): the gland shows clear signs of stimulation (colloid and follicular height). * Follicular cavities containing colloid, 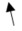 Follicular cells (thyrocytes), 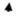 Endothelial cells (blood capillaries), - - - Epithelial height.

The lizard *Podarcis siculus* has a thyroid gland positioned transversely about halfway down the trachea; it has a ribbon-like structure formed by follicles that are connected by connective tissue containing blood vessels. Each follicle is formed by an epithelium, composed of thyrocytes that surround a cavity containing a medium-sized colloidal mass [31]. The thyroid gland of lizards exhibits a marked annual cycle, characterised by a functional stasis that begins in autumn and becomes complete stasis in December–January and a resumption of activity in spring with a maximum activity in May–June [31]. During the experimental period, control samples showed maximum thyroid stimulation, revealed by a very high follicular epithelium (11.1 ± 0.04 μm) and a reduced follicular diameter of 265 ± 26 μm (Table 1). Cylindrical thyrocytes have a prominent continuous epithelium; chromophobe droplets are visible in the colloid (Figure 5A).

The thyroid gland’s morphology was found to increase in response to different concentrations of NPY (1.25–2.50–3.75–6.25 nmol/100 g body wt) over various time frames (2–24 h). We examined the effect of NPY on follicular epithelium height and follicle diameter (both in μm) and the presence of colloid resorption vacuoles, which are important markers of morphological alterations to the thyroid gland. As shown in Figure 5B, the thyroid glands of lizards that were treated with one injection of NPY at the minimum dose (1.25 nmol/100 g body weight) and sacrificed 24 h after the injection appeared to have a follicular epithelium height that was quite similar to that of the control specimens (11.3 ± 0.51 μm) and a follicular diameter of 266 ± 25 μm (Table 1). The thyrocytes still had a cubic shape, and the colloid showed rare reabsorption vacuoles compared to the controls. With increasing dose of NPY administered (2.50 nmol/100 g body wt) both after 2 h and after 24 h (Figure 5C), the follicular epithelium increased (12.3 ± 0.25 μm) and the follicle diameter decreased (246 ± 30 μm (Table 1). Simultaneously, numerous resorption vacuoles in the colloid were observed at the periphery and increased vascularization in the thyroid (Figure 5C).

The highest stimulation was obtained with the highest doses of NPY (3.75–6.25 nmol/100 g body wt) but mainly with the highest dose (6.25 nmol/100 g body wt) after 24 h from administration (Figure 5D,E). In fact, in lizards treated with a dose of 3.75 nmol/100 g body wt and sacrificed after 24 h, the follicles were irregular in shape with a very high epithelium (16.5 ± 0.25 μm, *p* < 0.001) and roundish nuclei at the base of the follicular cells. The colloid filled up the follicular lumen, showing numerous reabsorption vacuoles (Figure 5D). In the group of lizards treated with a dose of 6.25 nmol/100 g body wt and sacrificed after 24 h (Figure 5E), there was a major stimulation of the thyroid gland, which revealed an increase in follicular epithelium (24.3 ± 0.25 μm, *p* < 0.001). Data about the height of follicular epithelium and the follicle diameter are shown in Table 1.

## 3. Discussion

The present study presents the first evidence of changes in thyroid gland morphophysiology after NPY administration to the wall lizard, *Podarcis siculus*. Our results show that NPY administration leads to a dose- and time-dependent differential regulation and metabolism of the thyroid axis. Lizards showed an increase in circulating levels of T_3_ and T_4_, suggesting an activation of the thyroid axis accompanied by a reduction in plasma levels of TRH and TSH, presumably due to the action of thyroid hormones at the pituitary level and/or to the inhibition of the signal at the hypothalamic level.

In this study, therefore, we addressed the causal link between stimulation of thyroid hormone synthesis and release, inhibition of TRH neurons, and suppression of the TSH during NPY administration; the relevance of NPY signaling in thyrocytes supports the important role of the paraventricular nucleus (PVN) in the regulation of the HPT axis, and sheds light on how NPY integrates thyroid morphofunctional signalling. Therefore, NPY administration in lizards allowed us to investigate how thyroid and PVN signaling pathways are integrated in reptiles similarly to mammalian vertebrates [37]. The hypothalamic periventricular nucleus (PVN) is a major metabolic centre, which includes neurons that are critical for appetite control and necessary for the proper regulation of the hypothalamic–pituitary–thyroid (HPT) axis. Several neuropeptides synthesized by periventricular neurons, such as alpha–melanocyte–stimulating hormone (α-MSH), neuropeptide Y (NPY), and agouti-related protein (AgRP), have been implicated in the regulation of the HPT axis in mammals [37].

In this study, we demonstrate that specific inhibition of TRH neurons in the PVN by administered NPY does not inactivate the entire HPT axis, given the stimulation of thyroid hormones, including the expression of T_3_ contents in the liver. Furthermore, the presence of NPY in the cell tips of thyrocytes and around follicles suggests that NPY may act on the thyroid gland by an autocrine and/or paracrine mechanism to stimulate synthesis and release of thyroid hormones, which in turn may inhibit TRH neurons projecting to the median eminence by inhibiting pituitary TSH levels. Thus, although NPY is widely expressed in the PVN, it is unclear which or how many of these neurons are required to mediate this effect of NPY on the thyroid gland in reptiles.

Furthermore, studies in mammalian vertebrates by several groups [26,38] have established that TRH neurons in the PVN also co-express NPY1 and 5 receptors; therefore, it is still unclear whether each TRH neuron that projects to the median eminence to regulate pituitary TSH levels has the capacity to respond to all of these signaling pathways simultaneously. Thus, PVN TRH neurons may initially not respond to NPY administration but instead may respond to the high TH levels observed after NPY administration, assuming that all hypophysiotropic TRH neurons express NPY receptors and TRb should therefore respond to all signals. Indeed, it is reasonable to propose that TRH neurons integrate this multitude of inputs, initiating a metabolic response through regulation of the HPT axis. Thus, suppression of TRH neurons after NPY administration might be a secondary regulatory step induced by negative feedback of thyroid hormones on the suppression of TSH release.

Further supporting the possible explanation of TRH suppression in the presence of high circulating thyroid hormone levels is the increased synthesis of hepatic T3 contents. Indeed, our results demonstrate that NPY administration increases the expression and activity of hepatic monodeiodinase 5′-T4 ORD (type II), increasing hepatic T_3_ production and further increasing the high circulating thyroid hormone levels.

Our results show a time- and dose-dependent regulation of the thyroid axis by NPY, as demonstrated by changes in plasma concentrations of TRH and TSH at central and peripheral levels with alteration of hepatic monodeiodinase type 2 (Dio2) activity.

Furthermore, the results obtained enable us to define a set of morphological characteristics induced by thyroid stimulation and shared by all animals treated with NPY, namely an increase in follicular epithelium height and highly vacuolised, compact colloid.

Changes in TH levels in response to NPY administration are likely to be species-specific and may depend on the animals’ metabolism, feeding habits, and reproductive seasonality. For instance, NPY reduces TH levels in rodents; however, northern elephant seal (*Mirounga angustirostris*) pups do not experience a reduction in plasma tT_3_, free T_3_, tT_4_, or free T_4_ levels. Instead, they upregulate peripheral monodeiodinase activation pathways [39]. This suggests that, in mammals, the thyroid axis may not be suppressed during NPY administration, a phenomenon that may be specific to animals that depend heavily on lipid metabolism [40].

In summary, the present work shows a direct and indirect relationship between NPY and the thyroid gland. In fact, the NPY/PVN circuit is directly responsible for the adaptive response of the HPT by inhibiting TRH neurons and thus the suppression of TSH, with a consequent increase in thyroid hormones and further indirect action due to the suppression of the hypothalamic–pituitary axis in response to high levels of thyroid hormones.

## 4. Materials and Methods

### 4.1. Animals and Experimental Procedures

We performed the animal experiments according to the ethical provisions imposed by the European Union. The National Committee of the Italian Ministry of Health permitted the in vivo experimentation and we organized the experiments to minimize the number of animals used.

Sexually mature male specimens of the *Podarcis siculus* lizard were captured in Campania, specifically in an area near the metropolitan city of Naples with the highest population density in Europe, in June when the thyroid gland is active [31]. Then, we selected ten lizards without significant differences in body mass and placed them in large soil-filled terraria containing heather and indoor subjected them to a photoperiod (11 h of daylight) and natural temperatures (15–24 °C). Moreover, they were fed honeycomb moth (*Galleria mellonella*) caterpillars daily, water was available ad libitum, and to reverse capture-related stress conditions, an acclimatization period of 15 days was allowed before starting the treatment [41].

All of the lizards used for analysis were about the same age and were weighed on a precision balance before and after the experimental tests. The animals were sacrificed by decapitation after deep anaesthesia with ketamine hydrochloride (Parke-Davis, Berlin, Germany) 325 µg/g of body weight [42,43,44].

For the choice of doses to be used, reference was made to the very few available experimental animal studies concerning the potentially adverse thyroid effects of neuropeptide Y, particularly in rats exposed to average daily doses of NPY in which there are signs of alterations on the hypothalamic–pituitary–thyroid axis.

During the experimental period, the lizards were divided into eight experimental groups (*n* = 10) according to the following scheme (n = number of samples used in each experiment):

**Control Group**: Untreated control lizards were intraperitoneally injected twice with 50 μL of corn oil. From this group, five lizards were sacrificed 2 h after the last injection and five 24 h after last injection.

**Group I**: Lizards were treated with one intraperitoneally injection (ip) of 1.25–2.50–3.75–6.25 nmol/g/body wt of Neuropeptide Y (CAS 90880-35-6 Merck KGaA, Darmstadt, Germany) dissolved in NaCl 0.75%, with an injection volume of 0.1 mL. Injections were between 8:00 and 8:30 a.m., and lizards were sacrificed 2 h after the last injection.

**Group II**: Lizards were treated with one ip injection of 1.25–2.50–3.75–6.25 nmol/g body wt of Neuropeptide Y (CAS 90880-35-6 Merck KGaA, Darmstadt, Germany) dissolved in NaCl 0.75%, with an injection volume of 0.1 mL. Injections were between 8:00 and 8:30 a.m., and lizards were sacrificed 24 h after the last injection.

### 4.2. Biochemical Analysis

#### 4.2.1. Plasma TRH (Thyrotropin-Releasing Hormone), TSH (Thyroid Stimulating Hormone) and Thyroid Hormones Assays

As reported in Sciarrillo et al. [43,44], the determination of TRH and TSH levels was performed using an immunoradiometric assay (IRMA). Serum samples and standards were added to anti-ligand-coated tubes. The tracer/capture reagent, a blend of ligand-tagged TSH-rabbit antibody and 125I-labelled (10 pCi) reagent, was then added to each tube. Calculations were performed using a cubic spline function with the zero standard as one of the standard points. The minimum detectable dose was 0.01 µIU/mL, with an accuracy close to 100%, and mean intra- and inter-assay variances of 5.0% and 7.5%, respectively. Cross-reactivity studies were performed using substances which could theoretically interfere with the performance of the assay. Cross-reactivity for FSH, hCG, and LH in the TSH IRMA was less than 0.001, so it was not considered in the data calculations [43,44].

Sample serum and standards were added to anti-ligand-coated tubes. The tracer/capture reagent, a blend of ligand-tagged TRH-rabbit antibody and 125I-labelled (10 pCi) material, was then added to each tube. A cubic spline function with the zero standard as one of the standard points was used for calculations. The minimum detectable dose was 0.01 µIU/mL, with an accuracy close to 100%, and mean intra- and inter-assay variances of 4.0% and 6.5%, respectively. Cross-reactivity studies were performed using substances which could theoretically interfere with the performance of the assay. Cross-reactivity for FSH, hCG, and LH in TRH IRMA was less than 0.001, so it was not considered in the data calculations [43,44].

T_3_ and T_4_ levels were determined using radioimmunoassay (RIA) (Byk-Sangtec Diagnostica, Dietzenbach, Germany) [43,44].

The T_3_ assay involved the addition of a measured amount of sample serum and standards to a tube that has been coated with anti-T_3_ rabbit antibody. This is done along with a trace amount of radioactively labelled T_3_ ([125I]-T_3_, 165 kBq; Byk-Sangtec Diagnostica, Dietzenbach, Germany) and a blocking agent (Tris buffered saline, 4 mM ANS, 6 mM sodium salicylate with 0.2% sodium azide as a preservative; Sigma Chemical Co., St. Louis, MO, USA). This is done to release T3 from serum binding proteins. The sensitivity was 0.1 ng/mL, with an accuracy of approximately 97%. The intra-assay variance ranged from 1.0% to 2.6% across 20 assays, while the inter-assay variance ranged from 3.9% to 5.7% across 12 assays [43,44].

For T_4_, an amount of serum sample and standards was added to a tube coated with anti-T_4_ rabbit antibody, alongside a trace amount of radioactively labelled T_4_ ([125I]-T4, 165 kBq; Byk-Sangtec Diagnostica) and a blocking agent (Tris-buffered saline containing 4 mM ANS, 6 mM sodium salicylate and 0.2% sodium azide as a preservative; Sigma-Aldrich, Milano, Italy). This releases T4 from serum binding proteins. The sensitivity was 0.45 ng/mL, with an accuracy close to 100%. The mean intra- and inter-assay coefficients of variation were 4.6% and 4.3%, respectively. Cross-reactivity for T_4_ in the T_3_ RIA (1.3%) and for T3 in the T4 RIA (0.1%) were not considered in the data calculations [43,44]. 

#### 4.2.2. Hepatic Thyroid Hormones and 5′ ORD (Type II) Monodeiodinase Activity

Livers were then removed and flushed with a buffer composed of MOPS and EDTA at a pH of 7.4. The levels of T3 and T4 in the liver tissue were determined using a radioimmunoassay (RIA) and expressed as nanograms per milligram of tissue (fresh weight) [43,44]. Enzyme activity is expressed as pM T3 per gram of liver per hour [43,44]. 

### 4.3. Histological Analysis

Thyroid glands were removed and immediately fixed in Bouin’s fixative and processed for light microscopy (LM). Serially cut paraffin sections (7 µm) were stained with Galgano stain and observation was performed using a Zeiss Axioskop microscope (Zeiss, Milano, Italy). The height of the follicular cells (µm) and the diameter of the follicles (µm) were measured in 30 cells every three slides and always on the second section of both normal and treated samples using a digital system of image (KS 300) (Zeiss, Milano, Italy) [31,42,43,44,45].

### 4.4. Statistical Analysis

The obtained data were averaged prior to calculating the experimental group mean and the standard error of the mean. As revealed by the χ^2^ test (chi-square test), data were not different from the normal distribution. The control and experimental data of all the groups were tested together for significance using two-way ANOVA, followed by Bonferroni’s for multi-group comparison using GraphPad Prism version 8.00 for Windows, GraphPad Software (La Jolla, CA, USA). Differences were considered significant at **** *p* < 0.001.

## 5. Conclusions

In conclusion, the present study is the first to report a dose-dependent variation of NPY administration in the wall lizard, where their maximum expression observed during thyroid gland recrudescence indicates the involvement of NPY in the HPT axis, confirmed by stimulation of thyroid hormone secretion and release and suppression of plasma TRH and TSH concentration. Our study provides preliminary evidence of a possible feedback loop between NPY and thyroid hormone metabolism in wall lizards, where NPY-mediated stimulation would lead to low TSH levels, which in turn would inhibit TRH neurons, resulting in inhibition of TRH levels. Thus, increased thyroid hormones may have an inhibitory effect on TRH in the diencephalon of wall lizards, thus giving rise to a negative feedback loop. The observations of the present study will provide the basis for future studies aimed at ascertaining these complex regulatory mechanisms.

## Figures and Tables

**Table 1:** Change in epithelial height (μm) and follicular diameter (μm) in the thyroid gland of *Podarcis siculus* subjected to one intraperitoneal injection of 1.25-, 2.50-, 3.75- or 6.25 nmol/100 g body wt of neuropeptide Y and sacrificed either 2 or 24 h after the last injection (* *p* <0.05, ** *p* < 0.01, *** *p* < 0.001, **** *p* < 0.0001 in comparison with the control). A more detailed description can be found in the text (see the Section 4).

Doses NPY (nmol g Body Weight)	Epithelial Height (μm)		Follicular Diameter (μm)	
	2 h *After Injection*	24 h *After Injection*	2 h *After Injection*	24 h *After Injection*
Control Group	11.1 ± 0.51		265 ± 25	
1.25	11.3 ± 0.51 *	11.5 ± 0.25 *	266 ± 25 *	269 ± 10 *
2.50	12.3 ± 0.25 **	12.8 ± 0.10 **	225 ± 30 **	219 ± 20 **
3.75	16.5 ± 0.25 ***	16.8 ± 0.05 ***	185 ± 20 ***	175 ± 15 ***
6.25	24.3 ± 0.25 ****	24.9 ± 0.05 ****	159 ± 15 ****	148 ± 15 ****

## Data Availability

The original contributions presented in this study are included in the article. Further inquiries can be directed to the corresponding author.
